# Examining the Relationship Between Executive Functions and Mentalizing Abilities of Patients With Borderline Personality Disorder

**DOI:** 10.3389/fpsyg.2020.01583

**Published:** 2020-07-14

**Authors:** Nándor Németh, Ágnes Péterfalvi, Boldizsár Czéh, Tamás Tényi, Maria Simon

**Affiliations:** ^1^Neurobiology of Stress Research Group, János Szentágothai Research Centre, University of Pécs, Pécs, Hungary; ^2^Department of Laboratory Medicine, Medical School, University of Pécs, Pécs, Hungary; ^3^Department of Psychiatry and Psychotherapy, Medical School, University of Pécs, Pécs, Hungary

**Keywords:** borderline personality disorder, mentalization, alexithymia, theory of mind, Reading the Mind in the Eyes Test, Faux Pas Test, executive functioning, symptom severity

## Abstract

Patients with borderline personality disorder (BPD) experience interpersonal dysfunctions; therefore, it is important to understand their social functioning and the confounding factors. We aimed to investigate the mentalizing abilities and executive functioning (EF) of BPD patients and healthy subjects and to determine the relative importance of BPD diagnosis and EF in predicting mentalizing abilities while controlling for general IQ and comorbid symptom severity. Self-oriented mentalizing (operationalized as emotional self-awareness/alexithymia), other-oriented mentalizing [defined as theory of mind (ToM)], and several EF domains were examined in 18 patients with BPD and 18 healthy individuals. Decoding and reasoning subprocesses of ToM were assessed by standard tasks (Reading the Mind in the Eyes Test and Faux Pas Test, respectively). Relative to controls, BPD patients exhibited significant impairments in emotional self-awareness and ToM reasoning; however, their ToM decoding did not differ. Multivariate regression analyses revealed that comorbid psychiatric symptoms were negative predictors of alexithymia and ToM decoding. Remarkably, the diagnosis of BPD was a positive predictor of ToM decoding but negatively influenced reasoning. Moreover, EF had no impact on alexithymia, while better IQ, and EF predicted superior ToM reasoning. Despite the small sample size, our results provide evidence that there is a dissociation between mental state decoding and reasoning in BPD. Comorbid psychiatric symptoms could be considered as significant negative confounds of self-awareness and ToM decoding in BPD patients. Conversely, the impairment of ToM reasoning was closely related to the diagnosis of BPD itself but not to the severity of the psychopathology.

## Introduction

Borderline personality disorder (BPD) is a psychiatric condition characterized by three symptom clusters including affective dysregulation, impulsivity, and disturbed relatedness (Sanislow et al., 2002). According to the mentalization-based model of BPD (Sharp and Kalpakci, 2015; Fonagy and Luyten, 2016), these features of BPD can be viewed as a consequence of impairments in the capacity to mentalize, i.e., to understand behavior in terms of underlying mental states. According to this theory, mentalization is defined as a multidimensional construct involving several dimensions and abilities. One of these dimensions relates to the objects of mentalizing: it can be directed either toward the mental states of the self or toward the mental states of others.

Impairment of self-oriented mentalizing can be manifested as low levels of emotional self-awareness or alexithymia (Choi-Kain and Gunderson, 2008). Alexithymia is a clinical condition characterized by an inability to identify and describe one’s own affective experiences (Taylor et al., 1997). Studies have found that borderline patients are more alexithymic than healthy controls (for a meta-analysis, see Derks et al., 2017) and reported relationships between BPD individuals’ alexithymic traits and the severity of their symptoms (e.g., Gaher et al., 2013; McMain et al., 2013). However, to date, no attention has been paid to the potential neurocognitive underpinnings of alexithymia in BPD.

Other-oriented mentalizing can be operationalized as theory of mind (ToM) (Choi-Kain and Gunderson, 2008), a social cognitive function by which we can attribute mental states, such as beliefs, intentions, and emotions, to others (Baron-Cohen et al., 1985). ToM is a multidimensional construct and consists of several subprocesses (Tager-Flusberg and Sullivan, 2000; Sabbagh, 2004). Mental state decoding is the social–perceptual aspect of ToM, which involves the ability to detect and discriminate others’ mental states based on their observable social behavior. Mental state reasoning implies the social–cognitive subcomponent, involving causal inferences and predictions about others’ mental states based on additional information sources including context and general social knowledge.

Findings on ToM performance in BPD indicate that the decoding and reasoning subprocesses of ToM may be unequally affected by the disorder. Several studies have found that BPD patients exhibited intact or even enhanced ability to decode others’ mental states based on facial expressions (Fertuck et al., 2009; Frick et al., 2012; Zabihzadeh et al., 2017). By contrast, other studies have shown that borderline patients perform worse than healthy controls on ToM reasoning tasks (Harari et al., 2010; Brüne et al., 2016), but the severity of their deficit is task dependent (Petersen et al., 2016). It has been suggested that BPD patients’ ToM impairment becomes apparent in more complex tasks that require contextual processing and the integration of multiple mental state perspectives (Baez et al., 2015; Petersen et al., 2016). This raises the possibility that the difficulties of BPD patients in ToM reasoning are not due to deficits in their basic ToM abilities but rather to deficits in neurocognitive skills, mainly in executive functioning.

Executive functioning (EF) refers to capabilities that enable flexible and goal-directed responses in novel or complex situations. Through the higher-order monitoring and regulation of cognitive subprocesses, EF plays an important role in the operation of many cognitive functions (Chan et al., 2008). The role of EF in mentalizing abilities is a widely investigated topic in both clinical and non-clinical samples. Regarding emotional awareness, it has been hypothesized that the cognitive systems that are responsible for the higher-level elaboration of emotional experiences are not specialized for emotional processing but rather implement domain-general executive functions (LeDoux, 2000; Lane and Garfield, 2005). This notion implies that executive dysfunction may cause disturbances in emotional self-awareness. Supporting this idea, several studies have found a relationship between poor performance on EF tasks and alexithymic symptoms (e.g., Henry et al., 2006; Santorelli and Ready, 2015).

Concerning the EF–ToM relationship, it has been suggested that these two abilities are implemented by two separate but interacting cognitive systems (Stone and Gerrans, 2006; Aboulafia-Brakha et al., 2011; Wade et al., 2018). According to this view, there are cognitive mechanisms specifically involved in the representation of mental states, but domain-general executive processes are required to efficiently manage and properly apply those representations in complex circumstances. In line with this assumption, many behavioral studies have demonstrated that performance on EF tests shows association with ToM performance, mainly in the case of those complex ToM tasks that have high cognitive load and contextual demands (Aboulafia-Brakha et al., 2011; Ahmed and Stephen Miller, 2011). These results suggest that EF is more strongly related to the reasoning aspect of ToM than to the decoding component.

There is a lack of research on the relationship between mentalizing abilities and EF in BPD. This limitation is particularly striking in studies that have demonstrated structural and functional abnormalities in frontal executive brain areas and impaired behavioral performance on executive tasks in borderline patients (Krause-Utz et al., 2014; McClure et al., 2016). Given this gap in the literature, the present study addressed two objectives. The first aim was to analyze simultaneously the mentalizing and executive profiles of BPD patients by comparing their performance to healthy individuals on tasks assessing different subdomains of mentalization and EF. Our second aim was to perform multivariate analyses to determine the relative importance of BPD diagnosis and EF in predicting alexithymia, as well as ToM performance while considering the potential effects of psychiatric symptom severity and general intelligence.

## Methods

### Participants

BPD patients (*N* = 18) were recruited from the Affective Disorder Unit of the Department of Psychiatry and Psychotherapy, University of Pécs. All patients fulfilled the Diagnostic and Statistical Manual of Mental Disorders, 5th edition (DSM-5) diagnostic criteria for BPD (American Psychiatric Association [APA], 2013). Exclusion criteria for the patient group were any other personality disorder, psychotic disorders, bipolar disorder, posttraumatic stress disorder, current substance use disorder, a history of head injury, neurological diseases, and intellectual disability. Healthy controls (HC, *N* = 18) were recruited through online advertisements. Exclusion criteria for controls included any mental disorder, a history of substance abuse, a history of neurological disorders, and head injury with loss of consciousness for more than 30 min, an IQ < 85, and any learning difficulties. All participants lived in the urban and suburban area of Pécs, were Caucasian, and native Hungarian speakers.

The diagnoses were established with structured clinical interviews (SCID-5-CV: First et al., 2016; SCID-5-PD: First et al., 2018). The severity of psychiatric symptoms was assessed with the Symptom Check List-90-Revised (SCL-90-R) questionnaire (Derogatis, 1977; Unoka et al., 2004), and the overall level of intelligence (IQ) was estimated with a four-subtest version of the Wechsler Adult Intelligence Scale-Revised (Kaufman et al., 1991). At the time of the investigations, 17 of the 18 patients were on psychotropic medication. Healthy controls were matched pairwise to the patients for sex, age (±4 years), education (±2 years), and IQ (±5 points). None of the healthy individuals took psychotropic medication. The clinical and demographic data are presented in Table 1.

**TABLE 1:** Demographic and clinical characteristics of the study samples.

	BPD (*n* = 18)		HC (*n* = 18)		*t*-value	*P*-value
**Demographics**						
Gender ratio (female/male)	17/1		17/1			
Age in years (mean ± SD)	34.72 ± 8.02		34.11 ± 9.39		0.210	0.835
Education level in years (mean ± SD)	12.78 ± 3.30		12.89 ± 2.78		–0.240	0.812
IQ estimate (mean ± SD)	109.79 ± 8.22		112.99 ± 8.60		–1.139	0.262
**Psychiatric symptom severity**						
SCL-90-R GSI (mean ± SD)	2.06 ± 0.66		0.40 ± 0.28		9.769	**<0.001**
SCL-90-R PST (mean ± SD)	66.94 ± 12.24		23.22 ± 12.42		10.639	**<0.001**
SCL-90-R PSDI (mean ± SD)	2.71 ± 0.47		1.48 ± 0.54		7.210	**<0.001**

	***n***	**%**	***n***	**%**	**Chisquare**	***P*-value**

**Current comorbid disorders**						
Depressive disorders	10	55.5	0	0	13.85	**<0.001**
Anxiety disorders	6	33.3	0	0	7.2	**<0.01**
Substance use disorders	5	27.7	0	0	5.81	**0.016**
Eating disorders	1	5.5	0	0	1.014	**0.31**
**Medications**						
Antidepressants	11	61.1				
Benzodiazepines	13	72.2				
Mood stabilizers	9	50				
Antipsychotics	16	88.8				

*Statistically significant *P*-values are written in bold. BPD, borderline personality disorder; HC, healthy controls; IQ, intelligence quotient; SCL-90-R, Symptom Check List-90-Revised; GSI, Global Severity Index; PST, Positive Symptom Total; PSDI, Positive Symptom Distress Index; SD, standard deviation.*

All subjects gave written informed consent, and the study was approved by the Research Ethics Committee of the Faculty of Humanities, University of Pécs (Ethical Approval No.: 2015/1).

### Instruments

#### Executive Function Tasks

Four subdomains of executive functioning (EF) were measured: (1) mental set shifting [with Wisconsin Card Sorting Test (WCST); Berg, 1948]; (2) working memory updating [with Listening Span Task (LST); Daneman and Blennerhassett, 1984; Janacsek et al., 2009]; (3) prepotent response inhibition [with Eriksen Flanker Task (FT); Eriksen and Schultz, 1979]; and (4) long-term memory access [with the Letter Fluency Task (LFT); see Strauss et al., 2006; Tánczos et al., 2014]. The WCST and the FT were computerized tasks taken from the Psychology Experiment Building Language (PEBL) test battery (Mueller and Piper, 2014). The EF variables of interest were the number of perseverative errors on the WCST, the number of words remembered in the LST, the interference time on the FT, and the number of words generated in the LFT. To get a global measure of executive functioning, we calculated an average *z* score from these four EF variables, which was converted into a *t* score (= composite EF score).

#### Mentalizing Tests

The level of emotional self-awareness/alexithymia was surveyed using the total scores of the 20-item self-report Toronto Alexithymia Scale (TAS-20; Bagby et al., 1994; Cserjési et al., 2007).

ToM capacities were examined with two standard ToM tasks. To measure ToM decoding ability, we used the Reading the Mind in the Eyes Test (RMET, Baron-Cohen et al., 2001; Ivády et al., 2007). This task is composed of 36 black-and-white photographs depicting the eye region. For each photograph, four mental state words were displayed, and the participants’ task was to decide which one best described what the person in the picture was feeling or thinking. As the RMET requires recognition of others’ mental states based on static and socially decontextualized perceptual stimuli, it does not necessitate contextual processing and complex inferences about mental states. Thus, the RMET is regarded as a prototypical task to measure the social–perceptual, decoding aspect of ToM (Sabbagh, 2004; Bora et al., 2006; Richman and Unoka, 2015).

ToM reasoning was assessed with the Faux Pas Test (FPT, Stone et al., 1998; Gál et al., 2011, 2014). This task consists of 20 short stories about different interpersonal situations that may or may not contain a social faux pas. After each story, participants were asked whether any of the story characters said something awkward. If participants said yes, further questions were raised regarding the characters’ cognitive and affective mental states. As a story-based verbal task, the FPT does not involve perceptual processing and requires causal inferences about the characters’ mental states on the basis of information provided by the contextual scenes and general social knowledge. Based on these features, the FPT is regarded as an appropriate task to investigate the social–cognitive, reasoning aspect of ToM (Wang et al., 2008; Thoma et al., 2013; Faiśca et al., 2016). (Detailed information about tests used in the study is reported in the Supplementary Material).

### Statistical Analysis

Between-group differences in demographic, clinical, neuropsychological, alexithymia, and ToM variables were analyzed using independent-samples *t* tests. For EF and mentalizing measures, we calculated Cohen’s *d* effect sizes. After the between-group comparisons, assumptions were tested, and multiple linear regression analyses were run in the whole sample. In the regression models, the total scores of TAS-20, RMET, and FPT were separately taken as dependent variables. BPD diagnosis (coded as a dummy variable: 0 = absence of the diagnosis, 1 = presence of diagnosis), SCL-90-R Global Severity Index (GSI), estimated IQ, as well as the composite EF scores were used as predictors in all models. To estimate the effect sizes of the predictors, Cohen’s *f*^2^ values were calculated. *P*-values (two-tailed) ≤ 0.05 were considered statistically significant.

## Results

### Between-Group Comparisons

The demographic and clinical features of BPD and HC groups are shown in Table 1. The groups were matched in terms of gender, age, education level, and estimated IQ. On the SCL-90-R questionnaire, the BPD group had significantly higher depression, anxiety, and global severity scores than the controls.

Group means and results of between-group comparisons for EF and mentalizing performances are presented in Table 2. There were no significant between-group differences in any EF domains. (We found a medium effect size for the composite EF and Inhibition scores, with a trend level significance of between-group difference for the latter one).

**TABLE 2:** Executive functions and mentalizing abilities in patients with borderline personality disorder (BPD) and healthy control (HC).

	BPD (*n* = 18)		HC (*n* = 18)		*t*-value	*P*-value	Cohen’s *d*
	
	Mean	SD	Mean	SD			
**Executive functions**							
Shifting (WCST perseverative errors)^a^	10.61	5.20	8.78	4.91	1.088	0.284	0.36
Updating (LST working memory span)	3.37	0.68	3.65	0.89	–1.046	0.303	–0.35
Inhibition (FT interference time)^a^	43.85	23.74	29.17	25.17	1.800	0.081	0.60
Access (LFT total words)	50.39	15.21	51.78	16.44	–0.263	0.794	–0.09
Composite executive function score	46.62	6.76	50.00	5.65	–1.629	0.113	–0.54
**Mentalizing**							
Alexithymia (TAS-20 total score)^a^	59.00	12.78	43.67	10.48	3.936	<0.001*	**1.31**
ToM decoding (RMET total score)	24.67	4.17	25.56	2.77	–0.753	0.457	–0.25
ToM reasoning (FPT total score)	27.78	5.94	31.89	4.55	–2.332	**0.026**	**−0.78**

Group means and between-group comparisons. BPD, borderline personality disorder; HC, healthy controls; WCST, Wisconsin Card Sorting Test; LST, Listening Span Task; FT, Flanker Task; LFT, Letter Fluency Task; TAS-20, Toronto Alexithymia Scale-20 items; RMET, Reading the Mind in the Eyes Test; FPT; Faux Pas Test; SD, standard deviation.^*a*^Higher scores indicate worse functioning. Statistically significant results are presented in bold. *Significant after Bonferroni correction. Cohen’s *d* values of 0.2, 0.5, and 0.8 represent small, medium, and large effect sizes, respectively.

#### Mentalizing Abilities

The BPD group had a significantly higher alexithymia score on the TAS-20 relative to the HC group (*P* < 0.001, Cohen’s *d* = 1.31). In our sample, ToM decoding (RMET) performances in the two groups did not significantly differ. However, the BDP group showed a significant impairment in ToM reasoning (*P* = 0.026, Cohen’s *d* = -0.78), as demonstrated by their lower mental state inference score on the FPT.

### Regression Analyses in the Whole Sample

#### Alexithymia

The multiple regression model predicting alexithymia was significant, explaining 56.9% of the variance in the TAS-20 scores. The diagnosis of BPD, the estimated IQ, and the composite EF score were non-significant predictors with small-to-medium effect sizes. General psychiatric symptom severity was the only significant predictor in the model (*P* = 0.002, Cohen’s *f*^2^ = 0.36) (Table 3).

**TABLE 3:** Multiple regression models for mentalizing abilities.

Variables	*B*	Std. Error	Beta	*t*-value	*P*-value	Cohen’s *f*^2^
**Alexithymia^a^**						
Constant	73.288	26.942		2.720	0.011	
BPD diagnosis	–5.479	6.694	–0.200	–0.818	0.419	−0.02
Symptom severity	12.797	3.805	0.904	3.363	**0.002**	**0.36**
IQ estimate	–0.308	0.233	–0.187	–1.322	0.196	−0.06
Executive functioning	4.271	3.098	0.196	1.379	0.178	0.06
**Theory of Mind decoding^b^**						
Constant	25.170	8.749		2.877	0.007	
BPD diagnosis	4.440	2.174	0.640	2.043	**0.050**	**0.14**
Symptom severity	–3.015	1.236	–0.841	–2.440	**0.021**	**−0.19**
IQ estimate	0.014	0.076	0.034	0.187	0.853	0.00
Executive functioning	0.805	1.006	0.146	0.800	0.430	0.02
**Theory of Mind reasoning^*c*^**						
Constant	1.224	11.743		0.104	0.918	
BPD diagnosis	–6.559	2.918	–0.592	–2.248	**0.032**	**−0.16**
Symptom severity	2.767	1.658	0.484	1.669	0.105	0.09
IQ estimate	0.262	0.101	0.394	2.577	**0.015**	**0.21**
Executive functioning	3.895	1.350	0.442	2.885	**0.007**	**0.27**

*Predictors of mentalizing abilities in the whole sample (*n* = 36). Cohen’s *f*^2^ values of 0.02, 0.15, and 0.35 represent small, medium and large effect sizes, respectively. Statistically significant results are presented in bold. BPD, borderline personality disorder; IQ, intelligence quotient. ^*a*^*F*(4,31) = 10.24, *P* < 0.001. ^*b*^*F*(4,31) = 3.19, *P* < 0.026. ^*c*^*F*(4,31) = 7.70, *P* < 0.001.*

#### ToM Decoding

The multiple regression model predicting ToM decoding accuracy was significant, accounting for 29.2% of the variance in the RMET scores. In this model, BPD diagnosis predicted significantly better performance on the RMET (*P* = 0.05, Cohen’s *f*^2^ = 0.14). However, greater psychiatric symptom severity was related to significantly worse performance (*P* = 0.021; Cohen’s *f*^2^ = -0.19). The cognitive variables and IQ were non-significant predictors with small effects (Table 3).

#### ToM Reasoning

The multiple regression model predicting ToM reasoning ability was significant, with 49.8% of the variance in the FPT scores accounted for by the predictors. BPD diagnosis was a significant negative predictor of FPT performance (*P* = 0.032, Cohen’s *f*^2^ = -0.16). Higher estimated IQ and composite EF scores predicted significantly better performance on the FP (*P* = 0.015, Cohen’s *f*^2^ = 0.21, and *P* = 0.007, *f*^2^ = 0.27, respectively). Only the general symptom severity was a non-significant predictor in this model (Table 3).

## Discussion

This is the first study to examine the relationship between EF, alexithymia, and ToM in BPD while simultaneously considering the confounding effects of psychiatric symptom severity and general IQ. Our results strengthen the notion that BPD patients’ mentalizing subdomains are dissociated: their self-awareness and ToM reasoning were impaired, while their ToM decoding was comparable with those of healthy controls. In a series of multiple regression models, we tested the relative predictive value of EF, IQ, the comorbid clinical symptoms, and the diagnosis of BPD on mentalizing capacities. Comorbid psychiatric symptoms had significantly negative relative importance while predicting self-awareness/alexithymia and ToM decoding. However, the diagnosis of BPD was proved to be a significant negative predictor of ToM reasoning but a positive predictor of decoding. EF and IQ positively influenced BPD patients’ ToM reasoning.

### The Executive and Mentalizing Profile of BPD

For assessing EF, we adopted theories about the fractionation of EF into different subcomponents (Miyake et al., 2000; Fisk and Sharp, 2004). There were no statistically significant between-group differences in any EF measures. However, BPD patients performed worse in the inhibition component of EF at a trend level significance (*P* = 0.081, with a medium effect size: Cohen’s *d* = 0.6). This trend-level between-group difference is in harmony with prior studies suggesting that deficits in response inhibition may be of central importance in BPD (Posner et al., 2002; Rentrop et al., 2008; Ruocco et al., 2012; van Dijk et al., 2014; Unoka and Richman, 2016). We can presume that the lack of significance was due to the low statistical power resulting from our small sample size.

Similarly to previous studies (for a review, see Derks et al., 2017), we found that BPD patients were significantly impaired relative to controls in their ability to mentalize (recognize and describe) their emotional states. Other-oriented mentalizing was operationalized in our study as ToM. The decoding and reasoning subcomponents of ToM were examined by prototypical tasks, the Reading the Mind in the Eyes Test, and the Faux Pas Test, respectively. Our results indicated that BPD patients’ ability to decode others’ mental states was preserved. By contrast, patients with BPD were impaired in their ability to reason about the mental states of others, evidenced by a large between-group difference in the number of correct mental state attributions on the Faux Pas Test. These findings replicated the results of several preceding studies and our recent meta-analysis that found similar performance on the RMET but substantially poorer performance on the Faux Pas Test in borderline patients compared to healthy controls (Baez et al., 2015; Petersen et al., 2016; Zabihzadeh et al., 2017; Németh et al., 2018). Our results endorse findings suggesting that the mentalizing profile in BPD is characterized by a dissociation between the decoding and the reasoning subprocesses of ToM.

### Factors Influencing Mentalizing Abilities

In our multiple regression model, neither general IQ nor global executive functioning was a significant predictor of alexithymia. Interestingly, not the diagnosis of BPD, but greater severity of comorbid psychiatric symptoms has been proven to be a relative predictor of a higher TAS-20 score. These findings are in line with prior studies (e.g., Loas et al., 2012; Pluta et al., 2018) demonstrating that borderline individuals are more alexithymic than healthy controls; however, this difference can mainly be explained by their comorbid clinical symptoms, especially by depression and anxiety. Although previous research has demonstrated a relationship between executive functioning and alexithymia in various clinical and non-clinical samples (Henry et al., 2006; Bogdanova et al., 2010; Koven and Thomas, 2010; Santorelli and Ready, 2015), our results suggest no relationship between these two abilities in BPD. Nevertheless, our study is the first that investigated this relationship in BPD; thus, further research with an extended number of cases is needed on this topic.

Remarkably, the multiple regression analysis predicting ToM decoding ability demonstrated opposing effects of BPD diagnosis and the severity of psychiatric symptoms. While BPD diagnosis predicted better performance on RMET, greater severity of coexisting psychiatric symptoms was associated with worse response accuracy. Previous studies using the RMET in borderline patients yielded inconsistent results, reporting reduced accuracy (Unoka et al., 2015; Van Heel et al., 2019), enhanced accuracy (Fertuck et al., 2009; Zabihzadeh et al., 2017), or no significant difference (Schilling et al., 2012; Baez et al., 2015) compared to healthy controls. Our findings suggest that the inconsistency of prior studies may be at least partly due to the confounding effect of the severity of psychiatric symptoms.

We found that BPD diagnosis was independently related to worse reasoning performance on the FPT, while psychiatric symptom severity was not a significant predictor in the model. However, both higher general IQ and better global EF were independently related to higher FPT scores. Contrary to our RMET results, here, we found that better EF was related to improved FPT performance. These findings suggest that these two ToM tasks may rely on different mechanisms. With its decontextualized stimuli, the RMET does not require contextual processing and complex reasoning processes. The FPT is a verbal task and requires causal inferences about mental states based on short stories in real-life social contexts. In the FPT, adequate mental state attribution depends not only on the ability to extract relevant information from the context but also on the ability to integrate representations of the characters’ mental states. Moreover, FPT also involves linguistic processing and other non-social cognitive skills and imposes additional cognitive load relative to the RMET. Our results suggest that this additional load uses up mainly executive function resources. These findings are in line with previous studies that examined the relationship between EF and ToM using RMET and FPT (e.g., Ahmed and Stephen Miller, 2011; Thoma et al., 2013; Baez et al., 2015; Torralva et al., 2015) and support the notion that the higher-order, reasoning aspect of ToM is more closely linked to domain-general cognitive abilities and prefrontal functioning than the lower-order, decoding component (Tager-Flusberg and Sullivan, 2000).

BPD diagnosis was also independently related to FPT performance in the multiple regression analysis. This negative effect of BPD remained significant in the model even after adjusting for general IQ, global EF, and psychiatric symptom severity. This suggests that mental state reasoning deficit might be a stable characteristic of the BPD. To date, only one study has examined the relationship between EF and ToM in BPD (Baez et al., 2015). Using similar ToM tasks, this research group found deficits both in EF and mental state reasoning in borderline patients. In their multivariate analysis, EF was significantly related to ToM reasoning performance, but BPD diagnosis was not a significant predictor of this ability, suggesting that mental state reasoning deficit is not a core feature of BPD, but is rather a consequence of executive dysfunction. Nevertheless, the small sample size is a major limitation for both studies; the contradictory relationship between EF and ToM in BPD deserves further examination.

### Limitations

The main limitation of our study was the low statistical power due to the small sample size. Thus, all of our findings must be treated as preliminary and should be replicated in larger samples. We should very carefully interpret our results especially those with EF. Executive dysfunction was suggested to play an important role in the pathomechanisms of BPD (Fertuck et al., 2006; Sebastian et al., 2014). A recent meta-analysis on neuropsychological functioning in BPD (Unoka and Richman, 2016) found a moderate effect size (Cohen’s *d* = -0.54) for EF impairment, which is the same as that on our composite EF scores. We can assume that our non-significant result in the between-group comparison of EF scores (*P* = 0.113) is largely due to the low number of cases.

Moreover, no clinical comparison group was included in the study; therefore, we did not investigate whether the detected mentalizing profile and its confounders are specific for BPD. BPD patients were recruited from the acute clinical setting. We did not examine demographic variables such as marital status and employment and did not follow-up on the sample to test how mentalizing abilities, cognitive functions, and comorbid clinical symptoms correlated with the demographic variables related to the functional outcome. Due to the high variability of comorbid psychiatric disorders and the psychotropic medications taken by BPD patients, it was not possible to form homogeneous subgroups to test the effect of these factors. Although a large proportion of our patients were on psychotropic medication (mainly on low-dose atypical antipsychotics), the impact of psychoactive drugs was not examined here. Finally, we only considered the severity of comorbid psychiatric symptoms measured by SCL-90; no other clinical questionnaires were applied.

## Conclusion

Acknowledging the limitations, the present study provides some important clues for therapy and future research on mentalizing abilities in patients with BPD. Our study presents further evidence that there is a dissociation between ToM decoding and reasoning abilities in BPD. Our results fit well to the theory of Fonagy and Bateman (2008): BPD patients who grow up in a non-reflecting, non-validating, and often abusing family environment develop an increased emotional vigilance to social stimuli, especially to those with negative emotional content. Nevertheless, BPD patients’ ToM abilities are just partially developed, since their reflexive awareness is low, and their mental state reasoning abilities are significantly impaired.

Based on our limited results, clinicians should carefully monitor BPD patients’ comorbid psychiatric symptoms and consider that comorbid symptoms can negatively impact the patients’ self-awareness and mental state decoding abilities. Conversely, impairment in mental state reasoning appears to be a core feature of BPD, but better IQ and EF can positively influence this deficit. However, regarding the low number of cases in our present study, further research is necessary to test our data in a larger sample.

## Data Availability Statement

The datasets generated for this study are available on request to the corresponding author.

## Ethics Statement

The studies involving human participants were reviewed and approved by Research Ethics Committee of the Faculty of Humanities, University of Pécs. The patients/participants provided their written informed consent to participate in this study.

## Author Contributions

NN and TT conceived the study and designed the experiments. NN collected and analyzed the data and drafted the manuscript. ÁP helped with the interpretation of data and writing of the manuscript. MS selected the patients, made the diagnosis, and revised the manuscript for publication. MS and BC raised funding and provided supervision and had helpful comments on the interpretation of the data. All authors contributed to the writing of the manuscript and/or revising it critically for important intellectual content. All authors approved the final version to be published and agreed to be accountable for all aspects of the work in ensuring that questions related to the accuracy or integrity of any part of the work are appropriately investigated and resolved.

## Conflict of Interest

The authors declare that the research was conducted in the absence of any commercial or financial relationships that could be construed as a potential conflict of interest.

## References

[B1] Aboulafia-Brakha T. Christe B. Martory M. D. Annoni J. M. (2011). Theory of mind tasks and executive functions: a systematic review of group studies in neurology. *J. Neuropsychol.* 5 39–55. 10.1348/174866410X533660 21366886

[B2] Ahmed F. S. Stephen Miller L. (2011). Executive function mechanisms of theory of mind. *J. Autism Dev. Disord.* 41 667–678. 10.1007/s10803-010-1087-7 20811770

[B3] American Psychiatric Association [APA] (2013). *Diagnostic and Statistical Manual of Mental Disorders*, 5th Edn Arlington, VA: American Psychiatric Association, 10.1176/appi.books.9780890425596

[B4] Baez S. Marengo J. Perez A. Huepe D. Font F. G. Rial V. (2015). Theory of mind and its relationship with executive functions and emotion recognition in borderline personality disorder. *J. Neuropsychol.* 9 203–218. 10.1111/jnp.12046 24766847

[B5] Bagby R. M. Parker J. D. A. Taylor G. J. (1994). The twenty-item Toronto alexithymia scale–I. Item selection and cross-validation of the factor structure. *J. Psychosom. Res.* 38 23–32. 10.1016/0022-3999(94)90005-1 8126686

[B6] Baron-Cohen S. Leslie A. M. Frith U. (1985). Does the autistic child have a “theory of mind”? *Cognition* 21 37–46. 10.1016/0010-0277(85)90022-8 2934210

[B7] Baron-Cohen S. Wheelwright S. Hill J. Raste Y. Plumb I. (2001). The “Reading the Mind in the Eyes” test revised version: a study with normal adults, and adults with Asperger syndrome or high-functioning autism. *J. Child Psychol. Psychiatry* 42 241–251. 10.1017/S0021963001006643 11280420

[B8] Berg E. A. (1948). A simple objective technique for measuring flexibility in thinking. *J. Gen. Psychol.* 39 15–22. 10.1080/00221309.1948.9918159 18889466

[B9] Bogdanova Y. Díaz-Santos M. Cronin-Golomb A. (2010). Neurocognitive correlates of alexithymia in asymptomatic individuals with HIV. *Neuropsychologia* 48 1295–1304. 10.1016/j.neuropsychologia.2009.12.033 20036267 PMC2843804

[B10] Bora E. Eryavuz A. Kayahan B. Sungu G. Veznedaroglu B. (2006). Social functioning, theory of mind and neurocognition in outpatients with schizophrenia; mental state decoding may be a better predictor of social functioning than mental state reasoning. *Psychiatry Res.* 145 95–103. 10.1016/j.psychres.2005.11.003 17074402

[B11] Brüne M. Walden S. Edel M.-A. Dimaggio G. (2016). Mentalization of complex emotions in borderline personality disorder: the impact of parenting and exposure to trauma on the performance in a novel cartoon-based task. *Compr. Psychiatry* 64 29–37. 10.1016/j.comppsych.2015.08.003 26350276

[B12] Chan R. C. K. Shum D. Toulopoulou T. Chen E. Y. H. (2008). Assessment of executive functions: review of instruments and identification of critical issues. *Arch. Clin. Neuropsychol.* 23 201–216. 10.1016/j.acn.2007.08.010 18096360

[B13] Choi-Kain L. W. Gunderson J. G. (2008). Mentalization: ontogeny, assessment, and application in the treatment of borderline personality disorder. *Am. J. Psychiatry* 165 1127–1135. 10.1176/appi.ajp.2008.07081360 18676591

[B14] Cserjési R. Luminet O. Lénárd L. (2007). Reliability and factor validity of the Hungarian translation of the Toronto alexithymia scale in undergraduate student samples. *Magy. Pszichol. Szle.* 62 355–368. 10.1556/MPSzle.62.2007.3.4

[B15] Daneman M. Blennerhassett A. (1984). How to assess the listening comprehension skills of prereaders. *J. Educ. Psychol.* 76 1372–1381. 10.1037/0022-0663.76.6.1372

[B16] Derks Y. P. M. J. Westerhof G. J. Bohlmeijer E. T. (2017). A meta-analysis on the association between emotional awareness and borderline personality pathology. *J. Pers. Disord.* 31 362–384. 10.1521/pedi_2016_30_257 27387060

[B17] Derogatis L. R. (1977). *Symptom Checklist 90, R-Version Manual I: Scoring, Administration and Procedures for the SCL-90.* Baltimore, MD: Johns Hopkins University Press.

[B18] Eriksen C. W. Schultz D. W. (1979). Information processing in visual search: a continuous flow conception and experimental results. *Percept. Psychophys.* 25 249–263. 10.3758/BF03198804 461085

[B19] Faiśca L. Afonseca S. Brüne M. Gonçalves G. Gomes A. Martins A. T. (2016). Portuguese adaptation of a Faux Pas test and a theory of mind picture stories task. *Psychopathology* 49 143–152. 10.1159/000444689 27271151

[B20] Fertuck E. A. Jekal A. Song I. Wyman B. Morris M. C. Wilson S. T. (2009). Enhanced reading the mind in the eyes’ in borderline personality disorder compared to healthy controls. *Psychol. Med.* 39 1979–1988. 10.1017/S003329170900600X 19460187 PMC3427787

[B21] Fertuck E. A. Lenzenweger M. F. Clarkin J. F. Hoermann S. Stanley B. (2006). Executive neurocognition, memory systems, and borderline personality disorder. *Clin. Psychol. Rev.* 26 346–375. 10.1016/j.cpr.2005.05.008 15992977

[B22] First M. B. Williams J. B. W. Benjamin L. S. Spitzer R. L. (2018). *Strukturált Klinikai Interjú a DSM-5^®^ Személyiségzavarok Vizsgálatára (SCID-5-PD).* Budapest: Oriold és Társai.

[B23] First M. B. Williams J. B. W. Karg R. S. Spitzer R. L. (2016). *Strukturált Klinikai Interjú a DSM-5^®^ Zavarok Felmérésére: Klinikai Változat (SCID-5-CV).* Budapest: Oriold és Társai.

[B24] Fisk J. E. Sharp C. A. (2004). Age-related impairment in executive functioning: updating, inhibition, shifting, and access. *J. Clin. Exp. Neuropsychol.* 26 874–890. 10.1080/13803390490510680 15742539

[B25] Fonagy P. Bateman A. (2008). The development of borderline personality disorder–a mentalizing model. *J. Pers. Disord.* 22 4–21. 10.1521/pedi.2008.22.1.4 18312120

[B26] Fonagy P. Luyten P. (2016). “A multilevel perspective on the development of borderline personality disorder,” in *Dev. Psychopathol.* (New York, NY: Willy) 3 726–792. 10.1002/9781119125556.devpsy317

[B27] Frick C. Lang S. Kotchoubey B. Sieswerda S. Dinu-Biringer R. Berger M. (2012). Hypersensitivity in borderline personality disorder during mindreading. *PLoS One* 7:e41650. 10.1371/journal.pone.0041650 22870240 PMC3411703

[B28] Gaher R. M. Hofman N. L. Simons J. S. Hunsaker R. (2013). Emotion regulation deficits as mediators between trauma exposure and borderline symptoms. *Cognit. Ther. Res.* 37 466–475. 10.1007/s10608-012-9515-y

[B29] Gál Z. Egyed K. Pászthy B. Németh D. (2011). Impaired theory of mind in anorexia nervosa. *Psychiatr. Hung.* 26 12–25. 21502668

[B30] Gál Z. Katona K. Janacsek K. Németh D. (2014). Theory of mind in offenders. *Pszichológia* 34 289–310. 10.1556/Pszicho.34.2014.3.5

[B31] Harari H. Shamay-Tsoory S. G. Ravid M. Levkovitz Y. (2010). Double dissociation between cognitive and affective empathy in borderline personality disorder. *Psychiatry Res.* 175 277–279. 10.1016/j.psychres.2009.03.002 20045198

[B32] Henry J. D. Phillips L. H. Crawford J. R. Theodorou G. Summers F. (2006). Cognitive and psychosocial correlates of alexithymia following traumatic brain injury. *Neuropsychologia* 44 62–72. 10.1016/j.neuropsychologia.2005.04.011 15896816

[B33] Ivády R. E. Takács B. Pléh C. (2007). “Tudatelmélet és idegen nyelvelsajátítás – valódi kapcsolat vagy városi legenda?,” in *Tudat és Elme*, eds Kampis G. Mund K. (Budapest: Typotex), 59–74.

[B34] Janacsek K. Tánczos T. Mészáros T. Németh D. (2009). The Hungarian version of listening span task. *Magy. Pszichol. Szle.* 64 385–406. 10.1556/MPSzle.64.2009.2.5

[B35] Kaufman A. S. Ishikuma T. Kaufman-Packer J. L. (1991). Amazingly short forms of the WAIS-R. *J. Psychoeduc. Assess.* 9 4–15. 10.1177/073428299100900101

[B36] Koven N. S. Thomas W. (2010). Mapping facets of alexithymia to executive dysfunction in daily life. *Pers. Individ. Dif.* 49 24–28. 10.1016/j.paid.2010.02.034

[B37] Krause-Utz A. Winter D. Niedtfeld I. Schmahl C. (2014). The latest neuroimaging findings in borderline personality disorder. *Curr. Psychiatry Rep.* 16:438. 10.1007/s11920-014-0438-z 24492919

[B38] Lane R. D. Garfield D. A. S. (2005). Becoming aware of feelings: integration of cognitive developmental, neuroscientific, and psychoanalytic perspectives. *Neuropsychoanalysis* 7 5–30. 10.1080/15294145.2005.10773468

[B39] LeDoux J. (2000). “Cognitive–emotional interactions: listen to the brain,” in *Cognitive Neuroscience of Emotion*, eds Lane R. D. Nadel L. (New York: Oxford University Press), 129–155.

[B40] Loas G. Speranza M. Pham-Scottez A. Perez-Diaz F. Corcos M. (2012). Alexithymia in adolescents with borderline personality disorder. *J. Psychosom. Res.* 72 147–152. 10.1016/j.jpsychores.2011.11.006 22281457

[B41] McClure G. Hawes D. J. Dadds M. R. (2016). Borderline personality disorder and neuropsychological measures of executive function: a systematic review. *Personal. Ment. 1008 Health* 10 43–57. 10.1002/pmh.1320 26381859

[B42] McMain S. Links P. S. Guimond T. Wnuk S. Eynan R. Bergmans Y. (2013). An exploratory study of the relationship between changes in emotion and cognitive processes and treatment outcome in borderline personality disorder. *Psychother. Res.* 23 658–673. 10.1080/10503307.2013.838653 24156526

[B43] Miyake A. Friedman N. P. Emerson M. J. Witzki A. H. Howerter A. Wager T. D. (2000). The unity and diversity of executive functions and their contributions to complex “frontal lobe” tasks: a latent variable analysis. *Cogn. Psychol.* 41 49–100. 10.1006/cogp.1999.0734 10945922

[B44] Mueller S. T. Piper B. J. (2014). The psychology experiment building language (PEBL) and PEBL test battery. *J. Neurosci. Methods* 222 250–259. 10.1016/j.jneumeth.2013.10.024 24269254 PMC3897935

[B45] Németh N. Mátrai P. Hegyi P. Czéh B. Czopf L. Hussain A. (2018). Theory of mind disturbances in borderline personality disorder: a meta-analysis. *Psychiatry Res.* 270 143–153. 10.1016/J.PSYCHRES.2018.08.049 30248485

[B46] Petersen R. Brakoulias V. Langdon R. (2016). An experimental investigation of mentalization ability in boderline personality disorder. *Compr. Psychiatry* 64 12–21. 10.1016/J.COMPPSYCH.2015.10.004 26608042

[B47] Pluta A. Kulesza M. Grzegorzewski P. Kucharska K. (2018). Assessing advanced theory of mind and alexithymia in patients suffering from enduring borderline personality disorder. *Psychiatry Res.* 261 436–441. 10.1016/j.psychres.2018.01.003 29360051

[B48] Posner M. I. Rothbart M. K. Vizueta N. Levy K. N. Evans D. E. Thomas K. M. (2002). Attentional mechanisms of borderline personality disorder. *Proc. Natl. Acad. Sci. USA.* 99 16366–16370. 10.1073/pnas.252644699 12456876 PMC138617

[B49] Rentrop M. Backenstrass M. Jaentsch B. Kaiser S. Roth A. Unger J. (2008). Response inhibition in borderline personality disorder: performance in a Go/Nogo task. *Psychopathology* 41 50–57. 10.1159/000110626 17975328

[B50] Richman M. J. Unoka Z. (2015). Mental state decoding impairment in major depression and borderline personality disorder: meta-analysis. *Br. J. Psychiatry* 207 483–489. 10.1192/bjp.bp.114.152108 26628692

[B51] Ruocco A. C. Laporte L. Russell J. Guttman H. Paris J. (2012). Response inhibition deficits in unaffected first-degree relatives of patients with borderline personality disorder. *Neuropsychology* 26 473–482. 10.1037/a0028715 22612574

[B52] Sabbagh M. A. (2004). Understanding orbitofrontal contributions to theory-of-mind reasoning: implications for autism. *Brain Cogn.* 55 209–219. 10.1016/J.BANDC.2003.04.002 15134854

[B53] Sanislow C. A. Grilo C. M. Morey L. C. Bender D. S. Skodol A. E. Gunderson J. G. (2002). Confirmatory factor analysis of DSM-IV criteria for borderline personality disorder: findings from the collaborative longitudinal personality disorders study. *Am. J. Psychiatry* 159 284–290. 10.1176/appi.ajp.159.2.284 11823272

[B54] Santorelli G. D. Ready R. E. (2015). Alexithymia and executive function in younger and older adults. *Clin. Neuropsychol.* 29 938–955. 10.1080/13854046.2015.1123296 26701062

[B55] Schilling L. Wingenfeld K. Löwe B. Moritz S. Terfehr K. Köther U. (2012). Normal mind-reading capacity but higher response confidence in borderline personality disorder patients. *Psychiatry Clin. Neurosci.* 66 322–327. 10.1111/j.1440-1819.2012.02334.x 22624737

[B56] Sebastian A. Jung P. Krause-Utz A. Lieb K. Schmahl C. Tüscher O. (2014). Frontal dysfunctions of impulse control – a systematic review in borderline personality disorder and attention-deficit/hyperactivity disorder. *Front. Hum. Neurosci.* 8:698. 10.3389/fnhum.2014.00698 25232313 PMC4153044

[B57] Sharp C. Kalpakci A. (2015). Mentalization in borderline personality disorder: from bench to bedside. personality disorders: theory, research, and treatment. *Personal. Disord.* 6 347–355. 10.1037/per0000106 26436578

[B58] Stone V. E. Baron-Cohen S. Knight R. T. (1998). Frontal lobe contributions to theory of mind. *J. Cogn. Neurosci.* 10 640–656. 10.1162/089892998562942 9802997

[B59] Stone V. E. Gerrans P. (2006). What’s domain-specific about theory of mind? *Soc. Neurosci.* 1 309–319. 10.1080/17470910601029221 18633796

[B60] Strauss E. Sherman E. M. S. Spreen O. (2006). *A Compendium of Neuropsychological Tests: Administration, Norms, and Commentary.* New York: Oxford University Press.

[B61] Tager-Flusberg H. Sullivan K. (2000). A componential view of theory of mind: evidence from Williams syndrome. *Cognition* 76 59–90. 10.1016/S0010-0277(00)00069-X 10822043

[B62] Tánczos T. Janacsek K. Németh D. (2014). Verbal fluency tasks I. Investigation of the Hungarian version of the letter fluency task between 5 and 89 years of age. *Psychiatr. Hung.* 29 158–180. 25041746

[B63] Taylor G. J. Bagby R. M. Parker J. D. A. Grotstein J. (1997). *Disorders of Affect Regulation: Alexithymia in Medical and Psychiatric Illness.* Cambridge: Cambridge University Press, 10.1017/CBO9780511526831

[B64] Thoma P. Winter N. Juckel G. Roser P. (2013). Mental state decoding and mental state reasoning in recently detoxified alcohol-dependent individuals. *Psychiatry Res.* 205 232–240. 10.1016/j.psychres.2012.08.042 22995039

[B65] Torralva T. Gleichgerrcht E. Ardila M. J. T. Roca M. Manes F. F. (2015). Differential cognitive and affective theory of mind abilities at mild and moderate stages of behavioral variant frontotemporal dementia. *Cogn. Behav. Neurol.* 28 63–70. 10.1097/WNN.0000000000000053 26102996

[B66] Unoka Z. Richman M. J. (2016). Neuropsychological deficits in BPD patients and the moderator effects of co-occurring mental disorders: a meta-analysis. *Clin. Psychol. Rev.* 44 1–12. 10.1016/j.cpr.2015.11.009 26708387

[B67] Unoka Z. Rózsa S. Kõ N. Kállai J. Fábián Á Simon L. (2004). Validity and reliability of the SCL-90 in a Hungarian population sample. *Psychiatr. Hung.* 19 235–243.

[B68] Unoka Z. S. Fogd D. Seres I. Kéri S. Csukly G. (2015). Early maladaptive schema-related impairment and co-occurring current major depressive episode-related enhancement of mental state decoding ability in borderline personality disorder. *J. Pers. Disord.* 29 145–162. 10.1521/pedi_2014_28_146 24932871

[B69] van Dijk F. Schellekens A. van den Broek P. Kan C. Verkes R.-J. Buitelaar J. (2014). Do cognitive measures of response inhibition differentiate between attention deficit/hyperactivity disorder and borderline personality disorder? *Psychiatry Res.* 215 733–739. 10.1016/j.psychres.2013.12.034 24418050

[B70] Van Heel M. Luyten P. De Meulemeester C. Vanwalleghem D. Vermote R. Lowyck B. (2019). Mentalizing based on external features in borderline personality disorder compared with healthy controls: the role of attachment dimensions and childhood trauma. *J. Pers. Disord.* 33 736–750. 10.1521/pedi_2019_33_373 30689514

[B71] Wade M. Prime H. Jenkins J. M. Yeates K. O. Williams T. Lee K. (2018). On the relation between theory of mind and executive functioning: a developmental cognitive neuroscience perspective. *Psychon. Bull. Rev.* 25 2119–2140. 10.3758/s13423-018-1459-0 29619648

[B72] Wang Y. Wang Y. Chen S. Zhu C. Wang K. (2008). Theory of mind disability in major depression with or without psychotic symptoms: a componential view. *Psychiatry Res.* 161 153–161. 10.1016/j.psychres.2007.07.018 18926572

[B73] Zabihzadeh A. Maleki G. Richman M. J. Hatami A. Alimardani Z. Heidari M. (2017). Affective and cognitive theory of mind in borderline personality disorder: the role of comorbid depression. *Psychiatry Res.* 257 144–149. 10.1016/J.PSYCHRES.2017.07.034 28755605

