# Supplementary material for: Examining the Relationship Between Executive Functions and Mentalizing Abilities of Patients With Borderline Personality Disorder
Source: Front Psychol. 2020 Jul 14;11:1583. doi: 10.3389/fpsyg.2020.01583 (PMC7372901; doi:10.3389/fpsyg.2020.01583)
Supplement: Supplementary file 1 [file Data_Sheet_1.docx]

Supplementary Material

[**Examining the relationship between executive functions and mentalizing abilities of patients with borderline personality disorder**](http://review.frontiersin.org/Document/DownloadPDF?articleId=525851&siteId=42&userId=721898&roleId=18)

[**Nándor Németh**](http://www.frontiersin.org/Community/WhosWhoActivity.aspx?sname=N%C3%A1ndorN%C3%A9meth&UID=731071) **^1^,** [**Ágnes Péterfalvi**](http://www.frontiersin.org/Community/WhosWhoActivity.aspx?sname=AgnesPeterfalvi&UID=734745) **^2^,** [**Boldizsár Czéh**](http://www.frontiersin.org/Community/WhosWhoActivity.aspx?sname=BoldizsarCzeh&UID=210497) **^1,2^,** [**Tamás Tényi**](http://www.frontiersin.org/Community/WhosWhoActivity.aspx?sname=Tam%C3%A1sT%C3%A9nyi&UID=469368) **^3^, Maria Simon* ^1,3^**

*** correspondence:**

Maria Simon, MD, PhD, E-mail: [simon.maria@pte.hu](mailto:simon.maria@pte.hu)

# Description of executive function and mentalizing tests

***Wisconsin Card Sorting Test***

Mental set shifting was examined with the computerized version of the Wisconsin Card Sorting Test (WCST; Berg, 1948) from the Psychology Experiment Building Language (PEBL) test battery (Mueller and Piper, 2014). The test consists of cards with different geometric shapes that vary according to their form (triangle, star, cross, or circle), color (red, blue, green, or yellow), and number (one, two, three, or four). Participants were instructed to match one card at a time to one of four stimulus cards without being informed of the sorting principle (that is according to form, color, or number of shapes). The participants' task was to find out the current method of sorting based on the provided feedback (“correct” or “incorrect”) and thus match correctly. After 10 correct sorts, the sorting rule changed without previous warnings. Perseverative errors occurred when participants persisted in using a previously successful sorting principle despite negative feedback indicating that the sorting rule had changed. We detected the number of perseverative errors as a measure of mental set shifting ability.

***Listening Span Task***

The Hungarian adaptation (Janacsek et al., 2009) of the Listening Span Task (LST; Daneman and Blennerhassett, 1984) was used to assess working memory updating. In this test, the experimenter read sentences aloud to the participants, who were asked to decide after each sentence whether the statement in the sentence was true or false. At the same time, they also had to memorize the final words of the sentences. The task consisted of three sequences of experimental blocks. Each sequence began with a two-sentence block, followed by a three-sentence block, a four-sentence block, and so on. At the end of a block, participants were required to recall the final words of the sentences in the order in which they had been presented. If participants succeeded in recalling the words in a given block, the experimenter proceeded to the next, longer block. Otherwise, the number of words correctly recalled in the previous block was recorded and a new sequence of blocks was presented. In this study, the mean of the three sequences of blocks was used to measure participants' working memory span.

***Flanker Task***

The ability to inhibit prepotent responses was investigated by the Eriksen Flanker Task (FT; Eriksen and Schultz, 1979) taken from the PEBL test battery (Mueller and Piper, 2014). In this computerized task, left- or right-pointing arrows were sequentially presented for 800 ms, with an inter-stimulus interval of 500 ms. During the inter-stimulus interval, a fixation cross appeared at the center of the screen to indicate the location of the target stimulus (a target arrow). In the congruent condition, the target arrow was surrounded by four flanker arrows, which pointed in the same direction as the central target (“← ← ← ← ←” or “→ → → → →”), in the incongruent condition the four flankers pointed in the opposite direction as the target (“← ← → ← ←” or “→ → ← → →”), and in the neutral condition, there were no distractors around the target arrow. The participants’ task was to focus on the direction of the central stimulus, and indicate as quickly as possible whether the target arrow pointed towards the left or right by pressing the left or right arrow keys on the keyboard, respectively. A total of 120 trials (40 per condition) were run in random order. Because incongruent trials generate conflict between two competing responses, they require an additional inhibitory control process that is not necessary for the congruent trials. This additional cognitive load typically results in slower responses and more errors (“conflict cost”) in incongruent compared to congruent trials. In the present study, the difference between the mean reaction times for incongruent and congruent conditions was used to assess the efficiency of response inhibition.

***Letter Fluency Task***

The speed and efficiency of access to long-term memory representations were examined by the Letter Fluency Task (LFT; see Strauss et al., 2006). In this test, participants had to orally generate as many distinct words as possible, beginning with a given letter (“K”, “T” and “A” in the Hungarian version; Tánczos et al., 2014) during a fixed period of time (60 sec per letter). They were also instructed not to provide proper names or repeat the same word, and not to use the same word with a different ending. We recorded the total number of words correctly produced across the three trials to measure the access component of executive functioning.

***Toronto Alexithymia Scale***

Alexithymia was surveyed using the Hungarian adaptation (Cserjési et al., 2007) of the 20-item self-report Toronto Alexithymia Scale (TAS-20; Bagby et al., 1994). The TAS-20 assesses three facets of the alexithymia construct: (1) difficulty in recognizing and understanding one's own feelings (Difficulty Identifying Feelings scale; e.g., “I am often confused about what emotion I am feeling”); (2) inability to communicate one's own feelings to others (Difficulty Describing Feelings scale; e.g., “It is difficult for me to find the right words for my feelings”); and (3) cognitive tendency to focus on external events rather than internal psychological experiences (Externally Oriented Thinking scale; e.g., “I prefer talking to people about their daily activities rather than their feelings”). Each item of the questionnaire is rated on a five-point Likert scale (1 = strongly disagree to 5 = strongly agree). The total score can range from 20 to 100 and the cut-off score > 60 indicates clinically significant alexithymia. The Hungarian validation of the instrument (Cserjési et al., 2007) reproduced the original structure with three subscales, and yielded similar internal consistencies with Cronbach's alphas of 0.77, 0.74, 0.76, and 0.57 for the total scale, and the three subscales, respectively. For this study, the total score of the test was used.

***Reading the Mind in Eyes Test***

To measure mental state decoding ability, we utilized the revised version of the Reading the Mind in Eyes Test (RMET; Baron-Cohen et al., 2001; Hungarian adaptation: Ivády et al., 2007). This task is composed of 36 black-and-white photographs depicting the eye region of different human faces expressing complex emotions or intentions. For each photograph, four mental state words (e.g., “Interested”, “Suspicious”, etc.) were displayed on the screen and participants’ task was to decide which one best described what the person in the picture was feeling or thinking. There was no time limit for answering. The total score was calculated by adding the number of correctly identified mental state terms for all items.

**Faux Pas Test**

The reasoning component of ToM was examined with the Faux Pas Test (Stone et al., 1998) in Hungarian translation, which had been successfully used in previous studies with healthy and clinical samples (Gál et al., 2011, 2014). In this task, the experimenter read to the participants 20 short stories about different interpersonal situations that may or may not have contained a social faux pas. A faux pas occurred in the stories when one of the protagonists said something without considering if it is something that another protagonist might not wanted to hear or know, and which had a negative emotional impact on the listener not intended by the speaker. Thus, in order to fully understand the faux pas, participants had to recognize that someone said something socially inappropriate, and simultaneously attribute two mental states to the protagonists. On the one hand, they had to appreciate that the speaker committed the faux pas because he or she did not know or realize something (cognitive mental state attribution), and, on the other hand, that the listener would feel hurt or insulted by the statement (affective mental state attribution). There were 10 faux pas stories and 10 control stories not containing a faux pas. After each story, participants were asked whether someone said something wrong or awkward (faux pas detection question). If they said yes, an emotion attribution question (“Why shouldn't he or she have said it or why was it awkward?”) and an intention attribution question (“Why do you think he or she said it?”) were raised. Participants received 1 point for each correctly answered question. For this study, the total score of the test was used.

**References**

Bagby, R. M., Parker, J. D. A., and Taylor, G. J. (1994). The twenty-item Toronto Alexithymia Scale—I. Item selection and cross-validation of the factor structure. *J. Psychosom. Res.* 38, 23–32. doi:10.1016/0022-3999(94)90005-1.

Baron-Cohen, S., Wheelwright, S., Hill, J., Raste, Y., and Plumb, I. (2001). The “Reading the Mind in the Eyes” Test revised version: a study with normal adults, and adults with Asperger syndrome or high-functioning autism. *J. Child Psychol. Psychiatry* 42, 241–251. doi:10.1017/S0021963001006643.

Berg, E. A. (1948). A simple objective technique for measuring flexibility in thinking. *J. Gen. Psychol.* 39, 15–22. doi:10.1080/00221309.1948.9918159.

Bora, E., Eryavuz, A., Kayahan, B., Sungu, G., and Veznedaroglu, B. (2006). Social functioning, theory of mind and neurocognition in outpatients with schizophrenia; mental state decoding may be a better predictor of social functioning than mental state reasoning. *Psychiatry Res.* 145, 95–103. doi:10.1016/j.psychres.2005.11.003.

Cserjési, R., Luminet, O., and Lénárd, L. (2007). Reliability and factor validity of the Hungarian translation of the Toronto Alexithymia Scale in undergraduate student samples. *Magy. Pszichológiai Szle.* 62, 355–368. doi:10.1556/MPSzle.62.2007.3.4.

Daneman, M., and Blennerhassett, A. (1984). How to assess the listening comprehension skills of prereaders. *J. Educ. Psychol.* 76, 1372–1381. doi:10.1037/0022-0663.76.6.1372.

Eriksen, C. W., and Schultz, D. W. (1979). Information processing in visual search: A continuous flow conception and experimental results. *Percept. Psychophys.* 25, 249–263. doi:10.3758/BF03198804.

Faiśca, L., Afonseca, S., Brüne, M., Gonçalves, G., Gomes, A., and Martins, A. T. (2016). Portuguese Adaptation of a Faux Pas Test and a Theory of Mind Picture Stories Task. *Psychopathology* 49, 143–152. doi:10.1159/000444689.

Gál, Z., Egyed, K., Pászthy, B., and Németh, D. (2011). Impaired theory of mind in anorexia nervosa. *Psychiatr. Hung.* 26, 12–25.

Gál, Z., Katona, K., Janacsek, K., and Németh, D. (2014). Theory of mind in offenders. *Pszichológia* 34, 289–310. doi:10.1556/Pszicho.34.2014.3.5.

Ivády, R. E., Takács, B., and Pléh, C. (2007). “Tudatelmélet és idegen nyelvelsajátítás – valódi kapcsolat vagy városi legenda?,” in *Tudat és Elme.*, eds. G. Kampis and K. Mund (Budapest: Typotex), 59−74.

Janacsek, K., Tánczos, T., Mészáros, T., and Németh, D. (2009). The Hungarian version of Listening Span task. *Magy. Pszichológiai Szle.* 64, 385–406. doi:10.1556/MPSzle.64.2009.2.5.

Mueller, S. T., and Piper, B. J. (2014). The Psychology Experiment Building Language (PEBL) and PEBL Test Battery. *J. Neurosci. Methods* 222, 250–9. doi:10.1016/j.jneumeth.2013.10.024.

Richman, M. J., and Unoka, Z. (2015). Mental state decoding impairment in major depression and borderline personality disorder: Meta-analysis. *Br. J. Psychiatry* 207, 483–489. doi:10.1192/bjp.bp.114.152108.

Sabbagh, M. A., Moulson, M. C., and Harkness, K. L. (2004). Neural correlates of mental state decoding in human adults: An event-related potential study. *J. Cogn. Neurosci.* 16, 415–426. doi:10.1162/089892904322926755.

Stone, V. E., Baron-Cohen, S., and Knight, R. T. (1998). Frontal lobe contributions to theory of mind. *J. Cogn. Neurosci.* 10, 640–656. doi:10.1162/089892998562942.

Strauss, E., Sherman, E. M. S., and Spreen, O. (2006). *A Compendium of Neuropsychological Tests: Administration, Norms, and Commentary*. New York: Oxford University Press.

Tánczos, T., Janacsek, K., and Németh, D. (2014). Verbal fluency tasks I. Investigation of the Hungarian version of the letter fluency task between 5 and 89 years of age. *Psychiatr. Hung.* 29, 158–80.

Thoma, P., Winter, N., Juckel, G., and Roser, P. (2013). Mental state decoding and mental state reasoning in recently detoxified alcohol-dependent individuals. *Psychiatry Res.* 205, 232–40. doi:10.1016/j.psychres.2012.08.042.

Wang, Y., Wang, Y., Chen, S., Zhu, C., and Wang, K. (2008). Theory of mind disability in major depression with or without psychotic symptoms: A componential view. *Psychiatry Res.* 161, 153–161. doi:10.1016/j.psychres.2007.07.018.
